# Dr. Roberton, on the Use of Cantharides

**Published:** 1810-04

**Authors:** 


### *To the Editors of the Medical and Phyfical Journal.*

Gentlemen,

THE more frequent opportunities I have of witnessing the diseases of the generative system, the more I am inclined to believe that it may be difficult to specify the extent of their ravages on any individual or class of organs belonging to the human body. The following case is not the only one, in both sexes, which I could adduce in proof of their widely devastating effects.

An unmarried gentleman, aged 30, was addicted to private indiscretion at a very early period, but being ignorant of the bad consequences of such practices, he continued this habit to excess for a considerable length of time. The first bad effects of these practices, which he did not then attribute to that cause, was frequent giddiness, attended with a sensation as if the earth, as he expressed himself, was sinking under him. Still, however, he did not re frain from his bad habits, and of course these symptoms gained ground. To these symptoms were added others; he became perpetually apprehensive and alarmed, he knew not for what, on the most trifling occasions; was almost constantly troubled with violent palpitation of his heart, with stinging or shooting pains across his chest; and on such occasions he experienced flushing of the face, and a most disagreeable heat all over his body, particularly in the palms of his hands and soles of his feet. His testes were lower than usual, particularly the left, and the spermatic chord was somewhat enlarged and very painful.

His

His mind now seemed to partake of the general disorder; he became aukward and stupid to a great degree; all his mental faculties suffered considerably, especially his memory, which, unless on some particular subjects, and those of the very simplest nature, almost entirely left him. He laboured under a continual fright, that at some period at no great distance, he would altogether lose his reason. What rendered his situation particularly distressing was, his being perfectly sensible of all his distresses, and of his mental deprivations as now related. He at length became alarmed for his safety, and was in continual fear that he would expire suddenly while in bed. It was not till this period, which was his 22d year, that he was resolved to lay aside all these bad practices, which he now concluded must have given origin to his present state. Soon after this, he first had connection with a female. He was now sensible of a great improvement in his health, the palpitation of his heart ceased, and he became somewhat cheerful in company; but still his mind was weak, unsettled, and so easily agitated, that it alone threw a damp on all the pleasures of life.

From his 22d till he arrived at his 26th year, he occasionnally used cold bathing, which he thought yielded him momentary relief, but produced no permanent good effect on his health. He at length, however, found that he had no inclination for sexual intercourse, nor were his powers in that way so vigorous as they had been for some time before. He now tried the effect of nourishing diet, and regularly used mineral waters for two seasons, which he thought of advantage to him.

About his 29th year he was greatly improved in his general health; his mind was at times cheerful, and even happy, more so than it had been from the age of fourteen or fifteen. In short, in respect to his feelings, he was quite a new creature.

One morning, about this period, while in bed, he had recourse, the first time for five or six years, to his old habit. He was immediately seized with a degree of stupidity and a kind of derangement, different and more distressing than he had ever before felt; a profuse sweat too instantly covered his whole body, so as to render his linen quite wet. He declared he never was in such a state of complete misery, and he earnestly wished that every moment might terminate his existence. He suffered a sort of delirium, yet was sensible of his state; he leaped out of bed and bathed his face in cold water, but this only seemed seemed if possible to increase his sufferings; he put on his clothes and stalked about the house like a person in despair; he drank several glasses of wine, then of ardent spirits, but they had no effect upon him. After this he went back to bed to endeavour to procure some sleep, and the spirits he had used assisting him, he slept about two hours. He was now somewhat refreshed, but still his mind was very much and strongly confused; he felt as if afraid of entirely losing his reasoning faculties: indeed, he assured me, that language could not convey his feelings at that time, and for nearly two years after, when he applied to me.

It is strange that this patient scarcely had an emission oftener than once in eight, ten, and sometimes fourteen days; yet it is evident that the effects produced on his general health, arose from the generative organs caused by the same means, which had in all my patients occasioned seminal evacuations.

Previous to his application to me, he had used bark and wine, and carbonate of iron, which had considerably improved his digestive organs, and even his general health, but still he was stupid, and, as he expressed himself, strange even to himself.

As there seemed to be no symptom or affection of any organ to contraindicate the administration of the cantharides, I did not hesitate to prescribe that medicine to this patient, according to the method formerly adverted to; at the same time, I recommended the use of the cold bath.

He took the medicine eight months and two weeks, before he received any thing more than temporary relief from it. He had frequently, for a day or two, experienced greater comfort than before its administration, but he uniformly relapsed into his old state. At the end, however, of the above period, he felt sensibly invigorated, his mind was more cheerful, and his testes werenot nearly so relaxed as formerly. He, however, continued the use of the medicine about seven months more, and he is now perfectly recovered. His mind is still occasionally gloomy, but that is of short duration, and, upon the whole, he enjoys his life with considerable comfort.

*Princes Street.*

